# Consumers' Perceptions of Healthy Food Availability in Online Food Delivery Applications (OFD Apps) and Its Association With Food Choices Among Public University Students in Malaysia

**DOI:** 10.3389/fnut.2021.674427

**Published:** 2021-08-23

**Authors:** Elisa Zhen Rong Eu, Mohd Jamil Sameeha

**Affiliations:** Nutritional Sciences Programme, Centre for Community Health Studies (ReaCH), Faculty of Health Sciences, Universiti Kebangsaan Malaysia, Kuala Lumpur, Malaysia

**Keywords:** consumers' perceptions, online food delivery applications, online food environment, food choices, healthy food availability

## Abstract

This retrospective cross-sectional study was conducted to study consumers' perceptions of healthy food availability in online food delivery applications (OFD apps) among public university students in Malaysia and its association with their food choices. A total of 290 subjects aged 19–29 years old were recruited from 20 public universities in Malaysia *via* snowball sampling. Data was collected through an online questionnaire which consisted of socio-demographic status, use of OFD apps (most frequently used brand, usage frequency, food choice, and expenditure per transaction), factors affecting food choice in OFD apps, consumers' perceptions of healthy food availability in OFD apps and recommendation for improvements. The most frequently used apps among the subjects was Food Panda (46.6%), however, majority of the subjects in this study (41.4%) rarely used OFD apps. Also, most of the subjects ordered unhealthy food (77.6%) and spent up to RM15–RM19 for each transaction (43.1%). There was no significant difference between the use of OFD apps and gender (*p* > 0.05). Among the five food choice motives, “price and convenience” motive was the most influencing food choice factor in OFD apps. Majority of the subjects (76.9%) had a negative perception of healthy food availability (variety, price, and quality of healthy food) in OFD apps. No significant association was found between consumers' perceptions of healthy food availability in OFD apps and their food choices made in OFD apps among the subjects in this study (*p* > 0.05). Also, majority (85.9%) responded they are keen to purchase healthy foods through OFD apps if they are given an option. However, most Malaysian public university students perceived that there were not much variety of healthy food, of good quality and affordable price, available in OFD apps. This finding suggests that the online food environment in Malaysia are perceived as unhealthy. Future studies can explore the online food environment particularly its impact on community health and well-being. Public health professionals and policymakers need to address the online food environment issues as part of the obesogenic food environment in Malaysia especially when OFD is one of the most convenient service in this country.

## Introduction

The Internet and e-commerce industry have been growing at its fastest speed over the recent years. There have been a lot of changes in the traditional performance of doing tasks and jobs, with the rise of e-commerce and in particular mobile e-commerce, including the food and beverages industry. One successful outcome of this butterfly effect is the online food delivery service, or commonly known as the OFD apps. OFD apps can be defined as mobile apps that smartphone users download and use as an innovative and convenient channel to access restaurants, view food menus, place food orders, and make payments without any physical interaction with restaurant staff (1). In contrast to the conventional food delivery service platforms where consumers make a reservation in advance, the modern delivery service platforms provide service immediately upon receiving the order (2). Top 10 leading OFD apps in 2018 includes Meituan Takeout, Eleme, Koubei, and Jingdong To Home from China, Grubhub, Ubers Eats, and DoorDash from United States, Deliveroo and Just Eat from United Kingdom, Deliveryhero from Germany, and Swiggy from India (2). Table 1 lists the top 10 leading OFD services around the world in 2018 adapted from Tong et al. (2).

**Table 1:** Top 10 leading OFD brands around the world in 2018 adapted from Tong et al. (2).

**Name**	**Origin**	**Launch time**	**Number of customers in millions (M)**
Meituan takeout	China	2010	More than 300 M
Eleme	China	2009	~170 M
Koubei	China	2013	~170 M
Jingdong to Home	China	2015	More than 70 M
Grubhub	U.S.	2004	~17.2 M
Uber Eats	U.S.	2014	~8.1 M
DoorDash	U.S.	2013	~15.5 M
Deliveroo	U.K.	2013	~6 M
Just Eat	U.K.	2001	~26.3 M
Deliveryhero	Germany	2011	More than 17 M
Swiggy	India	2014	~4 M

More than a fifth of the population in China has experience in using OFD apps (3). In China, the OFD apps market is growing rapidly and has now become a major platform to generate incomes and increase revenue (4). Meanwhile, in Malaysia, OFD service is considered new compared to other countries like China and most OFD services are concentrated in big cities such as Kuala Lumpur, Klang Valley, Penang, and Johor Bahru. This is because OFD services in Malaysia are still facing the challenge of location and coverage boundary (5). However, the market is also starting to grow, though slow but steadily. Based on Euromonitor International, the food delivery market in Malaysia has a value of RM253 million in 2014 and is expected to continue to grow at 11% per annum (6). In short, there are two models in this modern food delivery system (7). The first model is known as in-house food delivery service and the food delivery service is fully controlled by the restaurant itself, where food is ordered online, through a website or mobile application, and delivered directly by the restaurant. This category is largely comprised of fast food chains such as Pizza Hut and McDonalds. The second model is the third-party food delivery service. One feature that distinguishes the two models is that the third party is responsible for the logistics of food delivery but not the restaurant itself (8). Some third party OFD apps in Malaysia include Food Panda and Grab Food.

The use of OFD apps is getting trendier, especially among urbanites, due to its convenience as OFD services allow them to have fresh and healthy food at their offices or homes while they have the freedom to continue to work (5). During the COVID-19 pandemic recently, the use of OFD apps was at its peak and the advantages brought by this service was largely highlighted. The shutdown of all non-essential services and restriction of restaurants to takeout service, in response to the social distancing measures, spark surge in food delivery service (9). It is undeniable that the growth of the OFD market has not only led to extraordinary sales to restaurants (10) but also driven the worldwide economic growth with revenue expected to increase to UDS182.3 billion by 2024 (11). Yet, a concern is beginning to arise in line with the growth of OFD services that are so trendy in today's society, which is the adverse effects brought by the trend of OFD applications on consumer health. Besides, the potential of OFD services to influence the current effectiveness of public health nutrition and health policies remains unknown (12). Calorie dense foods are among the most popular choices in OFD apps (13, 14). These includes fast foods such as cheeseburger and fries, pizzas, nachos, cheesecake, baby back pork rib and chicken and waffle sliders.

The use of OFD service is becoming more prominent among the young adults (15), especially university students. A survey in 2019 on 1,000 university students in Nanjing, revealed that at least 71.5% of them had used OFD for at least 2 years and that 85.1% of them used OFD more than once a week (16). Studies have shown university students tend to adopt unhealthy eating practices and they are prone to poor nutritional status (17–19). Many college and university students have difficulty in following healthy eating habits (20). This may be because they are away from home and not used to living independently (21) or may be because of the busy learning schedule which caused them to take too little time to prepare their own food (22). As a result, their diet changes. Furthermore, with the existence of OFD apps, the consumers, in particular university students, are exposed to more unhealthy food choices and this increases their chances of consuming unhealthy foods through the digital ordering.

The FSA (Food Standards Agency United Kingdom) has defined food choice as the selection of foods for consumption, which results from the competing, reinforcing, and interacting influences of variety of factors. These range from the sensory, physiological, and psychological responses of individual consumers to the interactions between social, environmental, and economic influences, and include the variety of foods and the activities of the food industry to promote them (23). It is undeniable that the main determinant of food choice is hunger, but food choice is not only determined by the physiological needs alone, or better known as nutritional needs only. In fact, the gender, age, and level of education of the consumer, along with perceptions, emotional motivations, and selection of sources of information on healthy eating should also be considered (24).

Besides, food choices are largely dictated by their availability within one's environment (25). Food environment is defined as a collective of the environment, opportunities, physical, economic, policy and socio-cultural conditions that can influence food choices and individual nutritional status (26). In fact, unhealthy diet is fuelled by an unconducive food environment (26). Nonetheless, an individual's perception on the healthfulness of environment might encourage more or less healthful purchase (27). In the context of OFD apps, the healthiness of food made available on the consumers remains unknown. Despite the convenience, OFD services may be a channel for university students to practice unhealthy eating practices and such services can be seen as a threat to student health. Hence, this study aim to determine the perceptions of OFD apps consumers represented by Malaysian university students on the availability of healthy food in OFD apps and its association with their food choices.

## Materials and Methods

### Study Design and Sampling

This retrospective cross-sectional study was carried out to study consumers' perceptions of healthy food availability in online food delivery applications (OFD apps) among public university students and its association with their food choices. Data collection was done in August 2020 and subjects were recruited *via* snowball sampling from 20 public universities in Malaysia. Only those who met the inclusion criteria were chosen as subjects. The inclusion criteria were (i) the subject must be a Malaysian Bachelor degree student aged 19 years old and above and (ii) had experience using OFD apps to purchase foods and beverages for his or her own consumption. Those who (i) did not own any OFD apps accounts, (ii) cannot understand either Malay or English and (iii) did not stay in the hostel were excluded in this study.

All responses were given based on the experiences before the Movement Control Order. The Malaysian Government Movement Control Order, or commonly known as MCO, is a cordon sanitaire implemented as a preventive measure by the Malaysian government in response to the Covid-19 pandemic in the country on 18 March 2020 (28). During MCO, the nationwide higher education institutions were closed and students previously staying in hostel were required to leave the hostel and go back to their hometowns. Hence, the subjects were required to recall their use of OFD apps when they were still staying in the hostels before the pandemic happen while answering the online questionnaire. Subjects were given an online subject information sheet to read and understand thoroughly followed by a consent from *via* Google Form. Ethics approval was obtained from the Research Ethics Committee of Universiti Kebangsaan Malaysia (UKM PPI/111/8/JEP-2020-412).

### Questionnaire Design

The questionnaire was developed based on comprehensive literature reviews on related topics such as consumers' perceptions, healthy food availability and OFD apps. The questionnaire comprised of five sections with a total of 32 questions. Before data collection, a pre-test was conducted on 30 subjects to test the reliability of the questionnaire. The two scales used in the questionnaire were reliable where the Cronbach's Alpha reliability coefficient for questions in Section C and Section D were 0.925 and 0.846, respectively. The result of Cronbach's Alpha test with reliability coefficient of more than 0.70 showed that the questionnaire was reliable (29).

Section A was to collect data on socio-demographic background, including gender, age, ethnicity, university and monthly allowance. For the use of OFD apps (Section B), five questions were asked namely most frequently used OFD apps brand, OFD apps usage frequency, food choice in OFD apps and expenditure spent per transaction. Section C measured health-related and non-health-related factors affecting consumer food choice in OFD apps. This section was adapted from Ooi et al. (30). There were 16 statements asked with a five-point likert scale method where 1: “very not important,” 2: “not important,” 3: “slightly important,” 4: “important,” and 5: “very important.” Subsequently, the consumers' perceptions of healthy food availability in OFD apps (Section D) was measured *via* three questions: (1) wide selection, (2) high quality, and (3) affordable price using a five-point likert scale ranging from 1 (strongly disagree) to 5 (strongly agree). The questions in this section was adapted from Barnes et al. (27). For analysis purposes, scores were given to each of the point chosen [1 (strongly disagree) = 0%; 2 (disagree) = 1%; 3 (neutral) = 2%; 4 (agree) =3%; 5 (strongly agree) = 4%] and a summary score for overall healthy food availability perception was created by adding all the scores for the three questions. Summary scores ranging from 0 to 12 wherein scores of 8 and below are categorized as negative perception of healthy food availability and scores of 9 and above are categorized as positive perception of healthy food availability (27). Section E was to collect data on consumer's recommendations for possible improvements in OFD apps.

### Statistical Analysis

Data analysis was performed using the SPSS version 25. The socio-demographic status of the subjects, their use of OFD apps, factors affecting consumer food choice, perceptions of healthy food availability in OFD apps and recommendations for improvements were summarized descriptively. Comparison on the use of OFD apps between gender was done using two different inferential analysis. Pearson Chi-Square test was used to analyse nominal variables including the apps brands and food choices while Mann-Whitney test was used to analyse ordinal variables such as OFD apps usage frequency and expenditure per transaction. Lastly, Pearson Chi-Square test was also used to determine the association between consumers' perceptions of healthy food availability in OFD apps and the food choices of the subjects. Findings with a *p*-value equal or < 0.05 were considered to be statistically significant.

## Results

### Socio-Demographic Characteristics

Table 2 shows the socio-demographic characteristics of the subjects. A total of 290 university students were recruited from 20 public universities in Malaysia. On average, subjects were university students aged from19 to 29 years old and majority were female (56.2%). A total of 47.9% of the subjects were Chinese. Among 20 universities, Universiti Kebangsaan Malaysia (UKM) had the highest number of subject (45.2%). Besides, majority of the subjects (38.6%) reportedly received a monthly allowance of RM401–RM700 while only 11% reported that they received more than RM1001 every month.

**Table 2:** Socio-demographic Characteristics of Subject.

**Socio-demographic characteristics**	**Total subject (** ***n*** **= 290)**	
	**Frequency (*n*)**	**Percentage (%)**
**Gender**		
Male	127	43.8
Female	163	56.2
**Age**		
19–23 years old	268	92.4
24–29 years old	22	7.6
**Ethnicity**		
Malay	92	31.7
Chinese	139	47.9
Indian	27	9.3
Others^a^	32	11.0
**University**		
Universiti Malaya (UM)	28	9.7
Universiti Sains Malaysia (USM)	22	7.6
Universiti Kebangsaan Malaysia (UKM)	131	45.2
Universiti Putra Malaysia (UPM)	9	3.1
Universiti Teknologi Malaysia (UTM)	6	2.1
Universiti Utara Malaysia (UUM)	6	2.1
Universiti Islam Antarabangsa Malaysia (UIAM)	1	0.3
Universiti Malaysia Sarawak (UNIMAS)	28	9.7
Universiti Malaysia Sabah (UMS)	12	4.1
Universiti Pendidikan Sultan Idris (UPSI)	11	3.8
Universiti Teknologi MARA (UITM)	10	3.4
Universiti Sultan Zainal Abidin (UNISZA)	3	1.0
Universiti Malaysia Terengganu (UMT)	2	0.7
Universiti Sains Islam Malaysia (USIM)	1	0.3
Universiti Tun Hussien Onn Malaysia (UTHM)	5	1.7
Universiti Malaysia Pahang (UMP)	4	1.4
Universiti Malaysia Perlis (UNIMAP)	1	0.3
Universiti Malaysia Kelantan (UMK)	2	0.7
Universiti Pertahanan Nasional Malaysia (UPNM)	1	0.3
Universiti Teknologi Malaysia Melaka (UTEM)	7	2.4
**Monthly allowance**		
≤ RM400	103	35.5
RM401–RM700	112	38.6
RM701–RM1000	43	14.8
≥RM1001	32	11.0

a *Others include Sabah and Sarawak natives and Malaysian Siamese*.

### Use of OFD Apps

Table 3 shows the use of OFD apps of the subjects. Among the brands, the most frequently used OFD apps were Food Panda (46.6%) and Grab Food (41.7%). Surprisingly, majority of the subjects (41.3%) reported that they rarely use OFD apps to purchase food and beverages. However, 40.3% of the subjects also reported that they use only 1–3 times per month. When asked about food choice made in OFD apps, more than half of the subjects (77.6%) purchased unhealthy food more frequently. Next, 43.1% of the 290 subjects spent RM15–RM19 per transaction (including delivery fee) while only 3.4% reportedly spent more than RM30 for each transaction. There was no significant difference between the use of OFD and gender (*p* > 0.05).

**Table 3:** Use of OFD apps.

**Characteristics**	**Male (** ***n*** **= 127)** ***n*** **(%)**	**Female (** ***n*** **= 163)** ***n*** **(%)**	**Total subject (** ***n*** **= 290)** ***n*** **(%)**	***P*** **-value**
**Apps brands^a^**				0.164
Food Panda	66 (52.0)	69 (42.3)	135 (46.6)	
Grab Food	53 (41.7)	68 (41.7)	121(41.7)	
Dah Makan	4 (3.1)	15 (9.2)	19 (6.6)	
Delivery Eat	1 (0.8)	5 (3.1)	6 (2.1)	
Running Man	2 (1.6)	4 (2.5)	6 (2.1)	
Others	1 (0.8)	2 (1.2)	3 (1.0)	
**OFD apps usage frequency^b^**				0.620
Rarely	54 (42.5)	66 (40.5)	120 (41.4)	
1–3 times monthly	51 (40.2)	66 (40.5)	117 (40.3)	
1–2 times weekly	19 (15.0)	22 (13.5)	41 (14.1)	
3–4 times weekly	2 (1.6)	7 (4.3)	9 (3.1)	
5–6 times weekly	1 (0.8)	2 (1.2)	3 (1.0)	
≥1 times daily	0 (0.0)	0 (0.0)	0 (0.0)	
**Food choices^a^**				0.895
Healthy food	28 (22.0)	37 (22.7)	65 (22.4)	
Unhealthy food	99 (78.0)	126 (77.3)	225 (77.6)	
**Expenditure per transaction^b^**				0.971
< RM15	28 (22.0)	43 (26.4)	71 (24.5)	
RM15–RM19	60 (47.2)	65 (39.9)	125 (43.1)	
RM20–RM24	30 (23.6)	40 (24.5)	70 (24.1)	
RM25–RM29	5 (3.9)	9 (5.5)	14 (4.8)	
≥RM30	4 (3.1)	6 (3.7)	10 (3.4)	

a *Analysis using Pearson Chi-Square test*.

b *Analysis using Mann-Whitney test*.

### Factors Affecting Food Choice in OFD Apps

Table 4 shows the ranking of five food choice motives and the respective influencing factors according to mean scores. The “price and convenience” motive was the most influencing factor (4.29 ± 0.76) that affects subjects' food choices in OFD apps. Followed by the “mood and sensory attraction” motive with a mean score of 4.14 ± 0.79. Next, “media influence” and “peer influence” were in third and fourth place with mean scores that only differ slightly, 3.30 ± 1.07 and 3.30 ± 1.03, respectively. With a mean score of 3.19 ± 1.00, the “health and nutrition knowledge” motive was the least influencing food choice factor.

**Table 4:** Factors affecting food choice in OFD apps.

**Ranking**	**Food choice motive**	**Mean score (mean ± s.d.)**
1	**Price and convenience** Take less time to prepare Can be delivered to where I live/study Is good value for money	4.29 ± 0.76
2	**Mood and sensory attraction** Taste good Looks nice Makes me feel good	4.14 ± 0.79
3	**Media influence** Is advertised in the media (television, radio, Internet etc.) Is the focus showed in advertisement Is as promoted in the advertisement in media	3.30 ± 1.07
4	**Peers influence** Is preferred by my friends Is recommended by my friends Is similar to those consumed by my friends	3.30 ± 1.03
5	**Health and nutrition knowledge** Contains natural ingredients Is nutritious and keeps me healthy Helps me control my weight Contains no artificial ingredients	3.19 ± 1.00

### Consumers' Perception of Healthy Food Availability in OFD Apps

Table 5 shows the consumers' perceptions of healthy food availability in OFD apps. Out of 290 subjects, most of them (76.9%) had negative perceptions on the healthy food available in OFD apps. On the other hands, 67 students perceived healthy food availability in OFD apps as positive.

**Table 5:** Consumers' perceptions on healthy food availability in OFD apps.

Consumers' perceptions^*^	**Total subject (** ***n*** **= 290)**	
	**Frequency (** ***n*** **)**	**Percentage (%)**
Positive perception	67	23.1
Negative perception	223	76.9

* *Consumers' perceptions refers to perceptions on healthy food availability based on three aspects namely variaty, price and quality of healthy food in OFD apps*.

Table 6 shows the subjects' responses to variety, quality and price of healthy food availability in OFD apps. Three statements were asked with a five-point Likert scale (1: “Strongly disagree,” 2: “Disagree,” 3: “Neutral,” 4: “Agree,” 5: “Strongly agree”). For ease of interpretation, subject responses were dichotomized into low agreement and high agreement. Majority of subjects had low agreement on the quality (59.7%) and price (61.4%) of healthy foods in OFD apps, except for the variety of healthy foods where half of the subjects had a high agreement (50.7%). This indicates that subjects' negative perceptions of healthy food availability may be due to unsatisfactory quality and/or higher prices of healthy food compared to unhealthy foods in OFD apps.

**Table 6:** Subjects' responses on healthy food availability in OFD apps based on variety, quality, and price.

**Aspect**	**Question**	**Subject's agreement**	
		**Low^a^** ***n*** **(%)**	**High^b^** ***n*** **(%)**
Variety	A wide selection of healthy food is available in OFD apps.	143 (49.3)	147 (50.7)
Quality	Healthy foods available in OFD apps are of high quality.	173 (59.7)	117 (40.3)
Price	Healthy foods available in OFD apps are affordable.	178 (61.4)	112 (38.6)

a *Low agreement includes reponses for “Strongly disagree,” “Disagree,” and “Nuetral.”*

b *High agreement includes reponses for “Strongly agree” and “Agree.”*

### Association Between Consumers' Perception and Their Food Choices in OFD Apps

Table 7 shows the association between consumers' perceptions of healthy food availability in OFD apps and their food choices. Majority of the subject (223 subjects) had a negative perceptions on the healthy food available in OFD apps. Among these subjects, most of them (78.9%) ordered more unhealthy food compared to healthy food. In contrary, out of 67 subjects who perceived positively, 73.1% of them reportedly ordered more unhealthy food compared to healthy food. Thus, both subjects with positive perception and negative perception had approximately the same percentage for unhealthy food choices. However, no significant association was found between consumers' perception of healthy food availability and their food choices in OFD apps (*p* > 0.05).

**Table 7:** Association between consumers' perceptions of healthy food availability in OFD apps and their food choices.

**Variable**	**Category**	**Consumers' perceptions of healthy food** **availability in OFD apps**		**Total** ***n*** **(%)**	***x*** ^**2**^ **value**	***P*** **-value**
		**Positive** ***n*** **(%)**	**Negative** ***n*** **(%)**			
Food choice	Healthy	18 (26.9)	47 (21.1)	65 (22.4)	0.993	0.319^*^
	Unhealthy	49 (73.1)	176 (78.9)	225 (77.6)		
	Total	67 (23.1)	223 (76.9)	290 (100.0)		

* *Analysis using Pearson Chi-Square test, there was no significant correlation (p > 0.05)*.

### Recommendation for Improvement

Table 8 shows the subjects' intentions to order healthy food through the OFD apps in the future. Overall, majority of subjects (85.9%) had intentions to order healthy food through OFD apps, although the findings of this study showed that the majority of subjects (76.9%) also had a negative perception of the availability of healthy food in OFD apps (Table 5).

**Table 8:** Subjects' intentions to order healthy food *via* OFD apps in the future.

**Intention to order healthy food**	**Frequency (*n*)**	**Percentage (%)**
Yes	249	85.9
No	2	0.7
Not sure	39	13.4

Table 9 shows the subjects' responses toward apps recommendations in order to promote healthy food choices. 91.7% of the subjects had a positive response when asked about the addition of nutritional information such as calories, nutrients, allergens, and others in the OFD apps. Other apps recommendations include carrying out promotions especially for healthy foods options (83.8%); complement each order with free fruits (71.4%); provide healthy eating tips such as recommended serving size, cooking and preparation methods in OFD apps (59.0%) and limit unhealthy food choices in OFD apps and replace with more healthy options (43.1%).

**Table 9:** Subjects' responses toward apps recommendations to promote healthier food choice.

**Response toward apps recommendations**	**Frequency (*n*)**	**Percentage (%)**
Additional nutrition information such as calories, nutrient, allergen etc	266	91.7
Carry out promotions specially for healthy foods option such as discount vouchers, free delivery etc	243	83.8
Complement each order with free fruits	207	71.4
Provide healthy eating tips such as recommended serving size, cooking and preparation methods etc in OFD apps	171	59.0
Limit unhealthy food choices in OFD apps and replace with more healthy options	125	43.1

## Discussion

Among the apps brands, Food Panda and Grab Food were more popular among the subjects. Similar finding was found in a study on the most used food apps by young workers adults in Shah Alam (31). The less popular apps brands such as Dah Makan, Delivery Eat, Running Man, and other brands may have limited delivery coverage area. For example, the delivery coverage area for Dah Makan apps is only in Kuala Lumpur and Selangor while for Lolol apps is only in the states of Melaka, Johor and Selangor. Lau and Ng's study in year 2019 (5) stated that OFD services in Malaysia is still facing the challenges of location and coverage boundary. Most of the subjects in this study reported either rarely using OFD apps or only used 1–3 times monthly and this is different from students in China where majority of the Chinese university students reported that they used OFD delivery services more than once a week (32). For food choices in OFD apps, majority of the subjects reported choosing unhealthy food more than healthy food. This is unsurprising as most types of food available in the delivery service are considered unhealthy (33). An example that can be illustrated here is the consumption of bubble milk tea ordered through Grab Food among Malaysians, which was three cups per person per month! (34).

Gender is one of the attributes of perceptual differences among consumers, especially in the online purchasing setting (35). However, in this study, there was no significant difference between the use ODF apps and gender. This finding was inconsistent with Bae and Lee's study in year 2011 (36) who found that female consumers have a higher risk perception of online purchases and this causes them to tend to hesitate when making online purchases. In addition, the study also found that online consumers' comments and reviews can effectively reduce the risk perception of female consumer and further attract them to buy online (36).

Based on the results, “price and convenience” motive was the most influencing food choice factor in OFD apps while “health and nutrition knowledge” motive was the least influencing food choice factor. This finding is relatively consistent with Brown et al. that stated university students tend to choose food according to convenience and time available, sensory attraction and food price as compared to the nutritional value of the food (37). Moreover, university students' food choices are not necessarily healthy even if they have adequate knowledge on nutritional requirements. In fact, the convenience and sensory attraction of food becomes their priority when making food choices (38). According to a study conducted on 150 pairs of married couples, food price is not considered to be an important factor in making food choices but they have evaluated religion, health and convenience as the three most important food choice factors in food selection (39).

Next, majority in this study had a negative perception on the healthy food available in OFD apps. The responses given by the subjects were based on their perceptions toward the healthy food availability in OFD apps from three aspects, which were variety, quality, and price. With regards to the variety of healthy foods available in OFD apps, studies have shown that most of the ordered foods were unhealthy. Due to diverse and competing food-delivery platforms, consumers have the potential to select healthy options when opting to use digital ordering (40). However, a report from DoorDash, a popular apps highlighted that American consumers' top ordered foods include cheese burgers and fries, pizza, nachos, and others (13). This indicates that calorie-dense food options are more popular to order through the OFD apps. In addition, Grubhub states that more than half of consumers use their apps to order fast foods (14). From these data, it is clear that unhealthy food choices especially fast food are more dominant in OFD apps compared to healthy foods. As for availability of healthy food in terms of quality, one study has showed that negative comments on ingredients reflect consumer concerns about the quality and safety of food sold by OFD apps (41). In addition, some American high school administrations have re-evaluated their polices on food delivery to school and even banned their students from using such service due to the concern on food safety, foodborne illness, and allergic reactions (42, 43). Finally, for the availability of healthy food in terms of price, studies have shown that most of the healthy food available in OFD apps were pricey. According to a report from Grubhub that was generated in year 2018, the average cost for a school lunch delivery service that provides food labeled “healthy,” “fresh,” or “organic” is $4 to $8 and this price is only for entrée meals (14). However, these prices are more expensive when compared to State School Nutrition data from 2017 highlighting lunch costs ranging from $2.48 to $2.74 per meal (44). This indicates that healthy food delivery may be available within an environment, however, it could be inaccessible due to the pricing matter.

This study found no significant association between consumers' perception of healthy food availability and their food choices in OFD apps. This is in contrary with previous studies which found otherwise. For example, there were some studies that stated the existence of a significant relationship between consumer perceptions and their food choices. For example, Barnes and his team found that those with positive perceptions on the neighborhood food environments are more likely to buy fruits and vegetables (28). Another study found that a positive perception on the food shopping environment is associated with higher intake of fruits and vegetables (45). In addition, consumers are willing to pay more for food that they consider healthy (46). These findings show that consumers, not just OFD apps consumers, may choose food based on how they perceived the particular food environment.

Most subject in this study was found keen to purchase healthy food *via* OFD apps in the future and most of them agreed that additional nutritional information can promote healthier food choice in OFD apps. Based on a study from Jo et al., it was found that after knowing the nutrient information, consumers are more willing to sacrifice sensory attraction for the sake of health (46). Although there are evidences showing the beneficial effect of caloric information on consumer food choices (47, 48), there are also some studies that did not find significant differences in consumer caloric intake between situations with and without caloric information (49, 50). This suggests that existence of nutritional information does not necessarily lead to healthy food choices.

There were several limitations in this study. Firstly, this was a retrospective study and responses given by the subjects when answering the online questionnaire were based on their memories. Secondly, due to the higher number of Chinese ethnic students and most subjects were UKM students, the data might be skewed to these factors. Besides, this study only recruited those studying Bachelor degree in public universities in Malaysia, hence the findings were unable to represent the whole population of university students in Malaysia. Also, no appropriate reference can be used to classify the food ordered by the subject through OFD apps as healthy or unhealthy. Therefore, bias may happen in food choice responses. This is because subjects' food choices were assessed in terms of their own perceptions but not assessed in terms of the type of food ordered whether it is healthy or not. In addition, the definition of nutritious food may also differ between different subjects.

## Conclusion

Most Malaysian public university students perceived that there were not much variety of healthy food, of good quality and affordable price, available in OFD apps. However, their perceptions toward the healthy food availability in OFD apps did not have any significant impact to them in choosing foods, either healthy or unhealthy, in OFD apps. Besides, majority of the participants had reflected interest in purchasing healthy foods *via* ODF apps if they are given an option. This finding can be used as a reference for OFD service owners to improve their existing food choices by providing more nutritious food. Other than introducing more nutritious food, pricing of food is also important especially when price is one of the most important factor in the food selection process of the subjects in this study.

In conclusion, the findings of this study suggests that the online food environment in Malaysia might be unhealthy. Future studies need to explore the online food environment particularly its impact to the community health and well-being, especially during the era of Covid-19 pandemic, where OFD apps are used more widely due to movement restriction orders. Public health professionals and policy makers need to address the online food environment issues as part of the obesogenic food environment in Malaysia especially when OFD service is one of the latest trend in this country.

## Data Availability Statement

The data that support the findings of this study are available from the authors, upon reasonable request.

## Ethics Statement

The studies involving human participants were reviewed and approved by Universiti Kebangsaan Malaysia Research Ethics Committee (Jawatankuasa Etika Penyelidikan Universiti Kebangsaan Malaysia). The patients/participants provided their written informed consent to participate in this study.

## Author Contributions

EE conceptualized and designed the study, conducted the study, data analysis and interpretation, prepared the draft of the manuscript, and reviewed the manuscript. MS assisted in conceptualizing and designing the study, assisted in drafting of the manuscript, and reviewed the manuscript. All authors contributed to the article and approved the submitted version.

## Conflict of Interest

The authors declare that the research was conducted in the absence of any commercial or financial relationships that could be construed as a potential conflict of interest.

## Publisher's Note

All claims expressed in this article are solely those of the authors and do not necessarily represent those of their affiliated organizations, or those of the publisher, the editors and the reviewers. Any product that may be evaluated in this article, or claim that may be made by its manufacturer, is not guaranteed or endorsed by the publisher.
